# Changes in Heart Rate Variability of Depressed Patients after Electroconvulsive Therapy

**DOI:** 10.1155/2012/794043

**Published:** 2012-08-27

**Authors:** Erica B. Royster, Lisa M. Trimble, George Cotsonis, Brian Schmotzer, Amita Manatunga, Natasha N. Rushing, Giuseppe Pagnoni, S. Freda Auyeung, Angelo R. Brown, Joel Schoenbeck, Smitha Murthy, William M. McDonald, Dominique L. Musselman

**Affiliations:** ^1^Department of Psychiatry & Behavioral Sciences, Emory University School of Medicine, Atlanta, GA 30322, USA; ^2^Department of Biostatistics and Bioinformatics, Emory University Rollins School of Public Health, Atlanta, GA 30322, USA; ^3^Center for Clinical Investigation, Case Western Reserve University School of Medicine, Cleveland, OH 44106, USA; ^4^Department of Biomedical Sciences, University of Modena and Reggio Emilia, 141100 Modena, Italy; ^5^GE Healthcare, Wauwatosa, WI 53226, USA; ^6^Department of Psychiatry & Behavioral Sciences, Mental Health Hospital Center, University of Miami Leonard M. Miller School of Medicine, Miami, FL 33136, USA

## Abstract

*Objective.* As few, small studies have examined the impact of electroconvulsive therapy (ECT) upon the heart rate variability of patients with major depressive disorder (MDD), we sought to confirm whether ECT-associated improvement in depressive symptoms would be associated with increases in HRV linear and nonlinear parameters. *Methods.* After providing consent, depressed study participants (*n* = 21) completed the Beck Depression Index (BDI), and 15-minute Holter monitor recordings, prior to their 1st and 6th ECT treatments. Holter recordings were analyzed for certain HRV indices: root mean square of successive differences (RMSSD), low-frequency component (LF)/high-frequency component (HF) and short-(SD1) versus long-term (SD2) HRV ratios. *Results.* There were no significant differences in the HRV indices of RMSDD, LF/HF, and SD1/SD2 between the patients who responded, and those who did not, to ECT. *Conclusion.* In the short term, there appear to be no significant improvement in HRV in ECT-treated patients whose depressive symptoms respond versus those who do not. Future studies will reveal whether diminished depressive symptoms with ECT are reliably associated with improved sympathetic/parasympathetic balance over the long-term, and whether acute changes in sympathetic/parasympathetic balance predict improved mental- and cardiac-related outcomes.

## 1. Introduction

 Alterations in autonomic nervous system activity have long been proposed as a potential mechanism contributing to the diminished survival of depressed patients with cardiovascular disease (CVD). Of the many arrhythmogenic factors, autonomic tone is the most difficult to measure [1]. The interplay and balance between sympathetic and parasympathetic input upon the cardiac pacemaker is reflected in the variability of the interbeat (R-to-R) interval duration within the normal sinus rhythm. A high degree of heart rate variability (HRV) is observed in healthy subjects with normal cardiac function; HRV can be significantly decreased in patients with severe coronary artery disease (CAD) or heart failure [2]. Heart rate variability analyses are traditionally performed in either the time domain (e.g., the standard deviation of interbeat intervals) or the frequency domain, giving in the latter case spectral measures of the interbeat interval time series (e.g., low-frequency power). Studies of HRV of depressed patients, without [3, 4] and with CAD [5, 6], have generally employed traditional HRV analyses, before and after a specific treatment for depression, be it psychotherapeutic [7, 8] or somatic [9–11]. More recently, the mathematical tools of nonlinear dynamics have been employed in HRV analysis to reveal alterations of HRV in depressed patients [12, 13]. Proponents of nonlinear dynamic techniques claim that such analyses may improve the ≤30% positive predictive value for cardiovascular outcomes currently obtained with classical time- and frequency-domain HRV analyses [14, 15]. 

 As three prior, small studies have shown decreased [16], or increased [17, 18], HRV in patients treated with electroconvulsive therapy (ECT), we sought to confirm whether ECT-associated improvement in depressive symptoms would be associated with an increases in HRV linear and nonlinear parameters of patients with major depressive disorder (MDD).

## 2. Methods

### 2.1. Subjects

 Psychiatric inpatients and outpatients between the ages of 18 and 90 years were recruited from the ECT service in the Department of Psychiatry and Behavioral Sciences based at Wesley Woods Geriatric Hospital in Atlanta, GA, USA. Patient recruitment occurred from 1999 to 2004. All patients fulfilled the Diagnostic and Statistical Manual of Mental Disorders, Fourth Edition criteria for Major Depressive Disorder (MDD) [19] and the American Psychiatric Association ECT task force for the recommendation of an acute course of ECT [20]. The Emory University Institutional Review Board approved this study, and written informed consent was obtained from patients in person or via their legal representative.

 Upon explanation of the study protocol, 100 patients provided their informed consent and underwent a semistructured psychiatric interview. Patients were excluded from analysis if they fulfilled any of the following: current alcohol or substance abuse or dependence, bipolar disorders (mania, hypomania, or cyclothymia), schizoaffective disorder, schizophrenia, diabetes, a myocardial infarction (MI) within the previous 6 months, and unstable or crescendo angina. Patients were also excluded if they had current untreated endocrine, cardiovascular, hematologic, hepatic, renal, or neurologic diseases. 

 Of those patients who provided signed informed consent (*n* = 100), patients were excluded due to lack of fulfillment of study inclusion criteria (*n* = 25), premature discontinuation of ECT (*n* = 6). Of the 69 patients remaining, 21 subjects were able to provide both Beck Depression Inventory (BDI) [21] scores and viable 15-minute heart rate variability recordings before their first ECT and sixth ECT treatment, the average number of treatments necessary for clinical improvement. Study patients were designated as “responders” to ECT if they reported a reduction of at least 50% from their baseline BDI scores. 

### 2.2. Procedures

 Prior to ECT treatment, the patients underwent a semistructured, psychiatric diagnostic interview; final psychiatric diagnoses were provided by consensus of a board-certified psychiatrist and the research team in accordance with Spitzer's procedure for longitudinal evaluation of all available data [22]. Medical diagnoses were based on medical history obtained from the patient self-report, medical records, physical examination, blood and urine laboratory analyses, and computed tomography of the brain. 

 Given the time constraints of our study population prior to their ECT treatments, and as HRV measurements have been routinely performed on ranging short-term (i.e., 15 minutes) to longer-term (i.e., 24 hours) EKG segments, EKG segments of 15 minutes in duration were acquired [2, 23], just prior to ECT-1 and ECT-6 between 7 and 10 AM while patients were stationary, and lying supine in a quiet, controlled setting 

 Recordings from the Marquette Series 8500 Holter monitor (GE Marquette Medical Systems, Milwaukee, WI, USA) were analyzed using a Marquette SXP Laser Holter scanner (software version 5.8) using standard techniques to label beats and edit artifacts. The ASCII files of R-R intervals in milliseconds were then transferred to a Sun SPARCstation computer for the characterization of linear (time and frequency domains) and nonlinear parameters of HRV (Table 1) using HRV Analysis software, version 2.0 (Biomedical Signal Analysis Group, Department of Applied Physics, University of Kuopio, Finland). In this software, the HRV spectrum is calculated with FFT-based Welch's periodogram method and with the AR method. Spectrum factorization in AR method is optional. In the Welch's periodogram method, the HRV sample is divided into overlapping segments. The spectrum is then obtained by averaging the spectra of these segments. This method decreases the variance of the FFT spectrum.

 ECT was administered as previously described [24, 25]. Patients continued their prescribed psychotropic and other medications [26–32] during their study participation. To minimize cognitive dysfunction, the average duration between ECT treatments was 48 to 72 hours; thus, the average duration from pre-ECT-1 testing to pre-ECT-6 testing was 12 days.

### 2.3. Statistical Analysis

 Chi-square tests (or Fisher's exact test for sparse data) and two-sample *t*-tests (or Wilcoxon two-sample tests for nonnormally distributed data) were used to determine significant differences in baseline demographic and clinical characteristics between excluded patients and included patients (ECT responders, ECT nonresponders). The same analysis was performed to determine differences in baseline demographic and clinical characteristics between ECT responders and ECT nonresponders. Change analysis for HRV variables (calculated as the HRV variable value at pre-ECT-6 minus pre-ECT-1) was performed using a two-sample *t*-test comparing the two groups of ECT-treated patients. 

## 3. Results

 As shown in Table 1, the excluded patients tended to be older and with a greater prevalence of men than the patients included in this study. The sample of included patients consisted largely of middle-aged, Caucasian women without a history of thrombovascular events. There were no differences between those who eventually responded to ECT versus those who did not, in the prevalence of traditional risk factors for CAD, for example, hypercholesterolemia or hypertension. As a group, the included patients experienced a significant decrease in their BDI scores from pre-ECT-1 (mean = 35.6 ± 10.3) to pre-ECT-6 (mean = 20.0 ± 10.5) (*P* < 0.001). Nearly half (47%) responded to ECT treatment, that is, experienced a reduction of at least 50% from their baseline BDI score.

 As shown in Table 2, many of study subjects were prescribed medications that could alter HRV at both ECT-1 and ECT-6 [26–35]. There was no difference in the prescription rates of the medication classes between ECT nonresponders and responders at either time point. 

**Figure 1:**
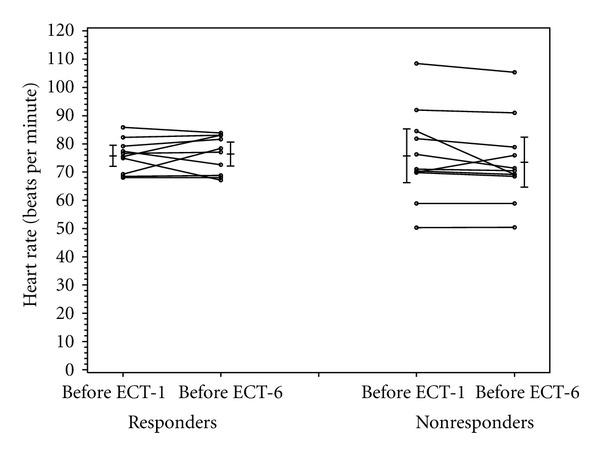
Heart rate in responders and nonresponders before ECT-1 and before ECT-6.

**Figure 2:**
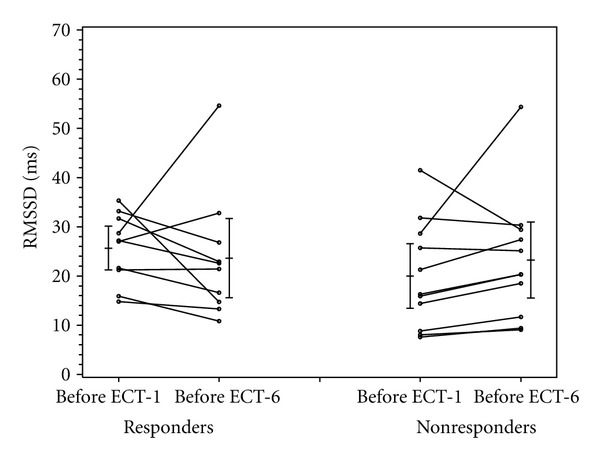
RMSSD in responders and nonresponders before ECT-1 and before ECT-6.

**Figure 3:**
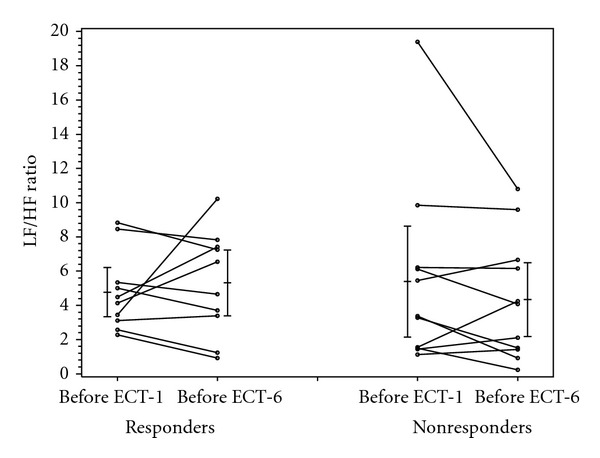
LF/HF ratio in responders and nonresponders before ECT-1 and before ECT-6.

**Figure 4:**
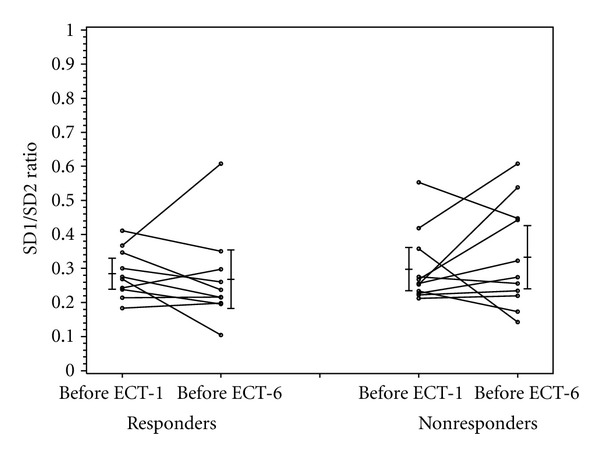
SD1/SD2 in responders and nonresponders before ECT-1 and before ECT-6.

 As shown in Table 3, the baseline BDI scores (before ECT-1) were not statistically different between patients who experienced a clinical response to ECT treatment (37.7 ± 9.8) and those who did not (33.7 ± 10.9) (*P* = 0.39). As the definition of “responder” was based on the change in BDI scores before ECT-1 and ECT-6, the decrease in BDI scores of the ECT responders (−25.2 ± 7.6) was significantly greater than that of the ECT nonresponders (−7 ± 7) (*P* < 0.001). At baseline, there were no significant differences in heart rate, RMSDD, LF/HF ratio, and SD1/SD2 between the ECT-responders and nonresponders (Figures 1–4). 

 Heart rate increased in the ECT responders and decreased in the nonresponders, though not significantly so (*P* = 0.21) (Figure 1). Similarly, there were no significant differences in the change of RMSDD, LF/HF, and SD1/SD2 between ECT responders and nonresponders (Figures 2–4). 

## 4. Conclusions

 Indeed, previous studies have documented that depression is reliably associated with diminished HRV [36]. This study sought to determine whether response to ECT would be associated with improvement in parameters of HRV of patients with depression whose depressive symptoms improved with ECT. Schultz and colleagues [16] were the first to document decreased vagal modulation in the time domain, that is, a decrease in RR interval, in middle-aged, depressed patients (aged 42 ± 12 years, *n* = 9) after ECT. Interestingly, the decrease in HF power in those patients correlated significantly with the magnitude of decrease in Hamilton Depression Rating Scale scores. In this study, there were no significant differences in the HRV parameters of RMSDD, LF/HF and SD1/SD2 between the patients who responded, and those who did not, to ECT. Although the changes in RSMDD were insignificant, RSMDD (which reflects vagus nerve-mediated autonomic control of the heart) appeared to decrease from pre-ECT-1 to pre-ECT-6 in the responders, and increase in the nonresponders (*P* = 0.26; Figure 2). Similarly congruent with the Schultz study, the LF/HF-ratio, which reflects the sympathovagal balance at the level of the sinus node, increased for the responders and decreased for the nonresponders (*P* = 0.21; Figure 3). 

 The two other existing studies of HRV in ECT-treated patients recruited older (70 ± 7 years, *n* = 10) [17] and middle-aged depressed patients (average age 45 years, *n* = 11) [18]. These groups documented increased vagal modulation as evidenced by increased HF (in normalized units) and a decreased LF/HF ratio [17], and SDNN (standard deviation of interbeat intervals) [18], respectively, in patients who respond to ECT. Nahshoni later used a nonlinear HRV parameter, PD2 (an estimate of RR intervals correlating with HF power), to document that the responders to ECT (*n* = 7; aged 70 ± 7 years) exhibited a significant increase in PD2 (*P* = 0.004), which tended to correlate with depressive symptom improvement (*r* = 0.73, *P* = 0.06). The discordant results of the above studies may be due, at least in part, to the effects of coadministered medications upon HRV, and to ECT's effects of the electrical charge upon the ANS during an acute administration, especially in patients with age-related declines in vagal modulation [20]. Indeed, ECT directly stimulates the vagus nerve and can cause asystole; within seconds of vagal stimulation, an adrenergic discharge related to the onset of a generalized seizure causes the release of epinephrine with hypertension, tachycardia, and the potential for arrhythmias or myocardial ischemia [37]. 

 A major limitation to our study, similar to prior studies, was its small size [16–18], which increases the possibility of committing a type II error in detecting a difference between the groups. Furthermore, larger sample size would have allowed adjustment in statistical models for medications which could potentially alter HRV. Recruitment of an age- and gender-matched, control group of depressed, ECT-treated patients without concomitant medications would have allowed us to understand the effects of medications upon HRV in this study population. 

 Another limitation of this study was the assessment of HRV before the first and sixth ECT sessions. In fact, remeasurement of HRV after termination of ECT or longer-term followup [17] would have allowed examination of whether HRV changes after ECT, either in a linear fashion or abruptly, when maximum improvement of depression may occur. Also arguable is whether 6 treatments comprised a “full course” of ECT, given that the HRV indices of ECT-nonresponders, and responders alike, might change over time. Nevertheless, this time point was selected, as six treatments are an average course of ECT at our center, which is administered using a consistent protocol measuring depressive symptoms with the BDI prior to each treatment, with RUL ECT at six times the seizure threshold. Of note is that the prior studies examining HRV in ECT-treated depressed patients have utilized either bilateral [16, 17] or unilateral [18] electrode placement. Bilateral ECT of our study population might have provided a more rapid treatment response [38], resulting in more patients designated as “responders” versus nonresponders. 

 Of the myriad HRV parameters, we selected the low-frequency (LF) parameter, and the LF/HF ratio, in order to examine the sympathetic contributions to modulation of HRV. Some interpret the LF parameter to reflect only sympathetic influence upon HRV, while others believe that LF reflects sympathetic and vagal modulation of HRV [2]. Thus, as an index of sympathetic-vagal balance, the LF/HF ratio has been controversial [39]. Indeed, the correlation between fluctuations in heart rate and LF/HF under resting conditions is generally weak [40, 41]. Nevertheless, based upon recommendations for HRV analyses of shorter-term data (less than 15 minutes in length) [2], we utilized time and frequency parameters with optimal reliability under resting conditions, that is, RMSDD and high frequency (HF), respectively. We offer that our use of short-duration HRV measurements under controlled conditions offered reasonable reliability and practical utility in this clinical population [42]. Other strengths of this study included the characterization of other traditional CAD factors, such as lack of exercise, hypercholesterolemia, gender, and prior thrombovascular events (Table 1), and tracking of all coadministered medications that could alter HRV (Table 2).

 Although ECT clearly benefits many patients, ECT may not improve HRV in the middle-aged, depressed patient without a history of clinically evident CAD [13], whose depressive symptoms respond to this somatic intervention. Future studies may reveal whether response to ECT is reliably associated with improved autonomic balance over the long-term, of elderly patients with a history of thrombovascular disease, and whether acute changes in HRV during ECT are associated with improved mental health and cardiac-related outcomes.

## Figures and Tables

**Table 1:** Demographic and medical characteristics of study population.

Characteristics	Excluded patients (*n* = 79)	Included patients (*n* = 21)	*P* value^a^	ECT Responder^b^ (*n* = 10)	ECT Nonresponder^c^ (*n* = 11)	*P* value^d^
Age (mean years)	56 ± 18	48 ± 18	0.06	48 ± 15	48 ± 20	0.95
Gender						
Female	47 (59%)	17 (81%)	0.06	9 (90%)	8 (73%)	0.59
Marital status						
Married	52 (69%)	12 (57%)	0.29	7 (70%)	5 (45%)	0.39
Race						
Caucasian	68 (88%)	21 (100%)		10 (100%)	11 (100%)	
African-American	7 (9%)	0 (0%)	0.26	0 (0%)	0 (0%)	n/a
Other	2 (3%)	0 (0%)		0 (0%)	0 (0%)	
Beck Depression Inventory score (mean ± SD)	32.5 ± 12.5	35.1 ± 10.5	0.38	37.7 ± 9.8	33.7 ± 10.9	0.29
Nicotine use	14 (18%)	4 (19%)	0.90	2 (20%)	2 (18%)	1.00
Hypertension^e^	29 (40%)	5 (24%)	0.18	2 (20%)	3 (27%)	1.00
Hypercholesterolemia^f^	32 (52%)	7 (39%)	0.34	4 (40%)	3 (38%)	1.00
Obesity^g^	40 (57%)	8 (38%)	0.13	4 (40%)	4 (36%)	1.00
Family history of premature CAD, MI, or stroke^h^	18 (30%)	4 (19%)	0.35	3 (30%)	1 (9%)	0.31
History of MI	5 (7%)	1 (5%)	0.71	0 (0%)	1 (9%)	1.00
Exercise^i^	23 (32%)	6 (29%)	0.77	2 (20%)	4 (36%)	0.64
History of stroke	3 (4%)	1 (5%)	1.00	0 (0%)	1 (9%)	1.00
History of TIA	3 (4%)	0 (0%)	1.00	0 (0%)	0 (0%)	n/a

ECT: electroconvulsive therapy; nicotine use: daily cigarette or pipe use; CAD: coronary artery disease; MI: myocardial infarction; TIA: transient ischemic attack; n/a: not applicable.

^a^ *P* values are based on comparisons between excluded and the 2 groups of included (nonresponder, responder) patients.

^b^ECT responders: study patients who experienced a decrease in Beck Depression Inventory (BDI) score of ≥50% from pre-ECT-1 BDI score.

^c^ECT nonresponders: study patients who experienced a decrease in Beck Depression Inventory (BDI) score of <50% from pre-ECT-1 BDI score.

^d^ *P* values are based on comparisons between ECT responders and ECT nonresponders.

^e^Hypertension was defined as a persistent diastolic blood pressure at or >90 mm Hg, or systolic blood pressure at 140–180 mm Hg.

^f^Hypercholesterolemia: total cholesterol of >200 ng/dL.

^g^Obesity as indicated by a body mass index of >25 (calculated as weight in kilograms divided by the square of height in meters).

^h^First-degree male relative <55 years and/or first-degree female relative <65 years with CAD, MI, or stroke.

^i^Exercise: self-report of regular aerobic exercise of 20-minute duration three times a week.

**Table 2:** Medications of study subjects with potential effects on heart rate variability before and during a course of electroconvulsive therapy.

		Before ECT-1		Before ECT-6	
		ECT Nonresponders (*n* = 11) (*n*, %)	ECT-Responders (*n* = 10) (*n*, %)	ECT Nonresponders (*n* = 11) (*n*, %)	ECT-Responders (*n* = 10) (*n*, %)
Medications that may increase HRV	Aminoketone antidepressant	0	1 (10%)	0	1 (10%)
	Antiarrhythmic	1 (9%)	0	1 (9%)	0
	Anticholesterol	2 (18%)	1 (10%)	2 (18%)	1 (10%)
	Benzodiazepine	6 (55%)	6 (60%)	4 (36%)	4 (40%)

Medications with variable effects on HRV	Beta blocker	2 (18%)	0	2 (17%)	1 (10%)
	Histamine-2 antagonist	3 (27%)	6 (60%)	5 (45%)	1 (10%)

Medications that may decrease HRV	Hormone replacements	3 (27%)	3 (30%)	3 (27%)	4 (40%)
	Smoking cessation	1 (9%)	1 (10%)	1 (9%)	0
	Tricyclic antidepressants	1 (9%)	1 (10%)	1 (9%)	0

ECT: electroconvulsive therapy; HRV: heart rate variability.

**Table 3:** Depression and heart rate variability measures of study participants before ECT-1 and before ECT-6.

Parameter	before ECT-1 (mean ± SD)	before ECT-6 (mean ± SD)	Change (mean ± SD)	*P* value^a^
BDI Score				
ECT responders	37.7 ± 9.8	12.5 ± 8.7	−25.2 ± 7.6	<0.0001
ECT nonresponders	33.7 ± 10.9	26.7 ± 6.9	−7.0 ± 7.4	
Heart Rate (bpm)				
ECT responders	75.7 ± 5.9	76.4 ± 6.7	0.6 ± 5.1	0.21
ECT nonresponders	75.8 ± 15.8	73.5 ± 14.8	−2.2 ± 5.1	
RMSDD (ms)				
Responders	25.7 ± 7.1	23.7 ± 12.7	−2.0 ± 11.9	0.26
Nonresponders	20.0 ± 10.9	23.3 ± 12.8	3.3 ± 8.9	
LF (ms^2^)				
ECT responders	918.5 ± 615.7	791.3 ± 589.0	−127 ± 562.8	0.88
ECT nonresponders	655.9 ± 804.5	481.8 ± 421.1	−174 ± 769.3	
HF (ms^2^)				
ECT responders	223.9 ± 160.5	246.4 ± 301.1	22.5 ± 294.0	0.98
ECT nonresponders	183.6 ± 234.8	202.8 ± 223.3	19.2 ± 204.4	
LF/HF				
ECT responders	4.77 ± 2.27	5.32 ± 3.03	0.55 ± 2.71	0.21
ECT nonresponders	5.39 ± 5.38	4.34 ± 3.58	−1.05 ± 2.93	
SD1				
ECT responders	18.2 ± 5.0	16.7 ± 9.0	−1.4 ± 8.4	0.26
ECT nonresponders	14.2 ± 7.7	16.5 ± 9.1	2.3 ± 6.4	
SD2				
ECT responders	66.2 ± 23.2	65.1 ± 20.1	−1.1 ± 23.6	0.88
ECT nonresponders	50.1 ± 29.1	50.7 ± 17.0	0.6 ± 25.9	
SD1/SD2				
ECT responders	0.28 ± 0.07	0.27 ± 0.14	−0.02 ± 0.11	0.37
ECT nonresponders	0.30 ± 0.11	0.33 ± 0.15	0.03 ± 0.14	

^a^ *P* values are based on comparisons of the change between ECT-1 and ECT-6 in ECT responders and ECT nonresponders.

ECT: electroconvulsive therapy; BDI: Beck Depression Inventory; RMSDD: root mean square of successive differences; LF: power in the low-frequency range (0.04–0.15 Hz); HF: power in the high-frequency range (0.15–0.4 Hz); SD1: standard deviation of the distances of points from the *y*-*x* axes.
